# Lung Cancer Screening Participation Among Indigenous Peoples Worldwide: A Systematic Review of Challenges and Opportunities

**DOI:** 10.1002/hpja.70001

**Published:** 2025-02-24

**Authors:** Sewunet Admasu Belachew, Habtamu Mellie Bizuayehu, Abbey Diaz, Shafkat Jahan, Sue Crengle, Kwun Fong, Gail Garvey

**Affiliations:** ^1^ First Nations Cancer and Wellbeing Research Program, School of Public Health The University of Queensland St Lucia Australia; ^2^ The Ngāi Tahu Māori Health Research Unit, Division of Health Sciences University of Otago Dunedin New Zealand; ^3^ Thoracic Research Centre The University of Queensland St Lucia Australia

**Keywords:** Australia, First Nations Peoples, lung cancer, review, screening

## Abstract

**Issue Addressed:**

Lung cancer screening (LCS) is crucial for Indigenous populations due to their higher lung cancer incidence rates and poorer outcomes. Despite efforts to establish LCS programmes, evidence on LCS cost‐effectiveness, participation rates, facilitators and barriers for Indigenous peoples remains limited. This systematic review aims to address this gap by reviewing available evidence.

**Methods:**

This systematic review conducted searches for relevant articles in PubMed, Scopus, CINAHL, Google Scholar and references/citations of included articles.

**Results:**

Fifteen out of 19 eligible studies were conducted in the USA, three in New Zealand and one in Canada, with 23 715 Indigenous participants in the 15 quantitative studies. New Zealand studies found that LCS is cost‐effective for Māori, while the participation rate for American Indian/Alaska Natives (4.7%) was lower than for White Americans (21.7%). Facilitators included positive views of LCS, trust in Indigenous‐centred care/providers, trusted invitations, family and community support, transportation or flexible scheduling, culturally competent navigators and detailed health education. Barriers included limited knowledge about LCS/eligibility criteria, fear of the screening process or cancer diagnosis, mistrust or negative experiences in healthcare, cost and time constraints, limited transportation/resources and non‐inclusive eligibility criteria.

**Conclusions:**

Further research is needed to understand the LCS among Indigenous peoples. Enhancing LCS participation requires leveraging positive experiences and addressing barriers with culturally tailored education and strategic resource allocation.

**So What?:**

For Australia and similar countries preparing for LCSPs, global evidence highlights the need for adequate resources, integration of Indigenous cultural practices and active involvement of Indigenous communities in programme planning.

**Abbreviations**
aOR: Adjusted odds ratio.
LCS: Lung cancer screening
LCSP: Lung cancer screening programme
LDCT: Low‐dose computed tomography

## 1 Introduction

Lung cancer is a leading cause of cancer‐related deaths worldwide [1] and disproportionately affects Indigenous peoples, who experience higher lung cancer incidence and mortality rates [2, 3]. For example, between 2009 and 2013, Aboriginal and Torres Strait Islander Peoples in Australia had an age‐standardised incidence rate of 85.2 cases per 100 000 and a mortality rate of 56.8 deaths per 100 000, both of which were twice the rates observed in non‐Indigenous populations [4]. Early detection via screening programmes can significantly improve outcomes [5, 6], particularly for Indigenous populations [7].

Global trials and high‐quality implementation studies have demonstrated that low‐dose computed tomography (LDCT) is cost‐effective for lung cancer screening (LCS) [8, 9]. Ten countries have initiated national or regional LCS programmes (LCSPs) [5], and several others are preparing for similar launches. For instance, Australia is set to begin its LCSP in July 2025 [5, 10]. LCSPs are crucial for Indigenous peoples due to their higher mortality rates from lung cancer. Despite ongoing efforts to establish LCSPs, concerns persist about meeting Indigenous peoples' needs, addressing existing cancer inequities and ensuring culturally safe and optimal LCS [11, 12, 13]. However, a world‐first, Indigenous‐led LCS trial for Māori participants has commenced in Aotearoa New Zealand, aiming to determine the impact of LCS invitation from primary care physicians versus centralised screening service on Māori screening participation providing policy‐relevant insights [14].

Promoting awareness of LCS and its benefits among Indigenous populations, as well as testing existing patient information and decision aids, is essential for culturally safe LCSPs [15, 16]. While not specific to Indigenous populations, a few studies and systematic reviews highlighted barriers and facilitators to LCS participation [17], including knowledge gaps, financial concerns and issues related to accessibility and availability, such as time constraints for both individuals and healthcare professionals [17, 18]. Involving multiple stakeholders in the formulation of LCS eligibility guidelines is crucial for ensuring adherence to LCSP and promoting inclusivity for underserved groups, including Indigenous peoples [19]. Despite these challenges, there is a lack of comprehensive systematic assessments of LCSP participation rates and the barriers and facilitators to participation for Indigenous peoples. This information is vital for the effective planning and implementation of LCSPs and addressing lung cancer disparities.

This systematic review examines global evidence regarding the barriers and facilitators affecting Indigenous peoples' participation in LCSPs. By synthesising the existing literature, it offers actionable insights for policymakers, healthcare providers and communities to improve equitable access and the effectiveness of LCSPs for Indigenous peoples. This review is timely, providing valuable information for the national implementation of LCSP in Australia and similar countries, with the goal of achieving a greater impact on Indigenous populations. Additionally, it highlights key areas for future research to support the successful implementation of these programmes for Indigenous populations.

## 2 Methods

### 2.1 Review Questions

This systematic review addresses the following research questions:

1. What is the evidence related to LCS cost‐effectiveness and participation rates for Indigenous populations?
2. What are the barriers and facilitators to LCS participation among Indigenous peoples?

### 2.2 Indigenous Terminology

Indigenous peoples are diverse, encompassing distinctive cultures, languages and rich cultural backgrounds. Various terms have been employed in the research to collectively refer to Indigenous peoples within specific regions or countries. These terms include American Indian, Alaska Native and Native Hawaiian in the United States of American (USA), Māori in Aotearoa New Zealand, Aboriginal and Torres Strait Islander in Australia, among others [20]. While we acknowledge that not all Indigenous peoples may resonate with the term ‘Indigenous’ for this review, the term ‘Indigenous peoples’ will be used respectfully, regardless of the country of origin, to describe the subjects of the included studies.

### 2.3 Search Strategy

This systematic review followed the Preferred Reporting Items for Systematic Reviews and Meta‐Analyses (PRISMA) guidelines [21]. A comprehensive search strategy incorporating keywords related to LCS and Indigenous peoples was developed and implemented to identify relevant studies in PubMed, CINAHIL and Scopus databases from their inception until the 5th of September 2024. An example of the search terms/strategies is described in Table S1. Additional searches were conducted on Google Scholar [22] and forward and backward citation searches of included articles using the Citationchaser tool [23].

### 2.4 Eligibility Criteria

Inclusion criteria: peer‐reviewed, original research articles published in English were included if they utilised quantitative, qualitative or mixed methods research design and reported on LCS cost‐effectiveness, barriers and facilitators, participation rates and/or eligibility guidelines for LCS in Indigenous populations from the perspectives of Indigenous peoples and/or other relevant health systems, health services, policy or programme development stakeholders. Studies describing LCS participation or completion rates or modelling of guideline eligibility criteria were included if they reported these as absolute measures (e.g., rates, percentages) or relative effect measures (e.g., odds ratio, relative risk), comparing outcomes for Indigenous people to non‐Indigenous people.

Exclusion criteria: case studies, case series, books, protocols, commentaries, editorial letters, conference abstracts, review papers and mass‐media publications. Additionally, studies that presented outcomes for Indigenous peoples combined with non‐Indigenous minority peoples, such as Asian/Pacific Islanders, were excluded.

In our review, participation rate refers to the proportion of LCS‐eligible individuals screened, completion rate refers to those screened after referral to LDCT and adherence rate refers to those completing recommended annual rescreening.

### 2.5 Study Selection and Critical Appraisal

**FIGURE 1:**
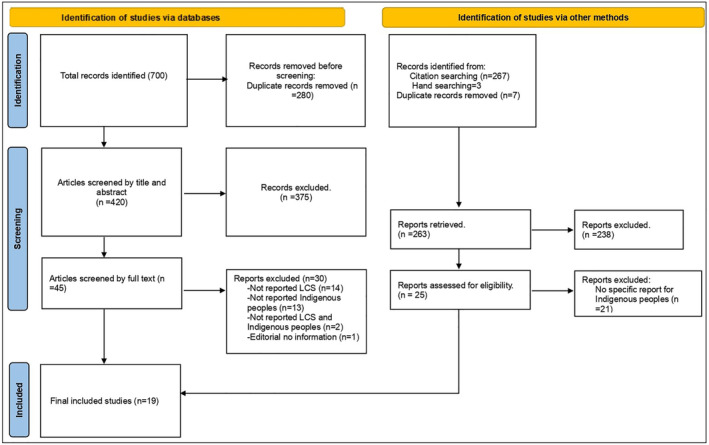
Flow diagram showing article selection.

The identified articles were exported to EndNote, and duplicates were removed. Two reviewers (SAB and HBM) independently screened titles, abstracts and full text (in that order) using the Rayyan software [24]. Any conflicts or disagreements between the reviewers at each stage were resolved through discussion (Figure 1). The included articles were independently appraised by two reviewers (SAB and HBM) using the Mixed Methods Appraisal Tool (MMAT) [25], designed to appraise reviews that include different study designs: qualitative, quantitative and mixed methods studies (Table S2).

### 2.6 Data Extraction and Synthesis

Two reviewers (SAB and HMB) performed data extraction using a customised Microsoft Excel spreadsheet. The data extraction focused on relevant information, encompassing study characteristics (i.e., study period, country, study population and sample size) and key findings, including barriers and facilitators to LCS.

A narrative synthesis approach was employed to systematically summarise relevant information (i.e., study characteristics, participation rates, barriers and facilitators to LCS among Indigenous peoples).

## 3 Results

### 3.1 Characteristics of Studies

We assessed a total of 970 studies for eligibility. Of these, 287 duplicate entries were removed, and a further 683 titles and abstracts were screened, and of 70 full texts examined, 51 were excluded based on the eligibility assessment. A total of 19 studies were included in the review (Figure 1).

Fifteen of the 19 included studies were quantitative designs, with 42 475 participants (23 715 Indigenous peoples and 401 035 non‐Indigenous peoples) [7, 19, 26, 27, 28, 29, 30, 31, 32, 33, 34, 35, 36, 37, 38]. Three qualitative studies and one mixed methods study were included [39, 40, 41, 42]. Fifteen of the 19 studies were conducted in the United States, three in New Zealand and one in Canada. Most studies (*n* = 12) were conducted at a sub‐national or provincial level (Table 1).

**TABLE 1:** Characteristics of the included studies.

ID	Period	Country^a^	Study design and data source^b^	Population^c^	Sample size
Anderson, 2023 [39]	2019–2020	USA (sub‐state)	Qualitative	Two types of participants were included: AI/AN patients who were current or previous commercial tobacco smokers, aged 45–79 years and attended Minnesota Urban Indigenous Community clinics and healthcare providers working in Minnesota Urban Indigenous Community clinics.	Nine key informant interviews among healthcare providers and four focus groups comprising 15 patients who were selected conveniently.
			A population‐based study with both focus groups and key informant interviews		
Colhoun, 2024 [40]	2019	New Zealand (sub‐national)	Qualitative	Māori in the Auckland and Waitematā regions, potentially eligible for LCS (current smokers or ex‐smokers who had quit within the previous 15 years and were aged between 50 and 75 years) and their whānau members.	Twenty‐one potentially eligible Māori (10 males and 11 females) and nine whānau members
Welch, 2024 [41]	2021	USA (National)	Qualitative	A convenience sample of American Indian/Alaska Natives adults, most of whom were aged 50 years and older. Participants were recruited in person at the Native Indian Council on Aging conference in Reno, Nevada, in August 2021.	52 American Indian
			Paper survey on demographics, smoking behaviour and knowledge of LCS was completed previously		1 Native Hawaiian or Pacific Islander
Pena, 2023 [36]	2021	USA (National)	Quantitative (Observational study)	AI/AN tribes in 35 US states	A total of 594 federally recognised AI/AN tribe in 35 US states met the study inclusion criteria.
Tsosie, 2024 [42]	2022–2023	USA (sub‐state)	Mixed‐methods study of qualitative + complementary survey	AI/AN people who were aged ≥ 40 and had ≥ 10‐year history of commercial cigarette smoking.	45 AI/AN participants
Dignan, 2024 [33]	2018–2022	USA (sub‐national)	Quantitative	High‐risk American Indian smokers living in four regions in Western South Dakota	A total of 962 community participants completed surveys at the education sessions. Of the 306 completing surveys at the 6‐month follow‐up.
					From July 2018 to June 2022, 2829 patients obtained an LDCT at screening centres participating in this investigation
Jaine, 2018 [34]	2011	New Zealand (national)	Quantitative (cost‐effectiveness analysis of a low‐dose computed tomography screening programme for lung cancer from the health system's perspective)	Adults aged 55–74 years who have 30 pack‐years smoking history and currently smoke or have quit within the past E15 years.	All (*N* = 52 704) non‐Māori (*N* = 45 645) Māori (*N* = 7059)
Choi, 2023 [32]	1993–1996 with follow‐up to 2018	USA (state)	Retrospective cohort study	Adults aged 45–75 years enrolled from the populations of California and Hawaii	Total (*N* = 105 261)
			A self‐reported questionnaire linked to SEER		Native Hawaiian/Other Pacific Islander (*n* = 8368)
Kim, 2022 [35]	2015–2017 with follow‐up to 2019	USA (National)	Retrospective cohort study	Adults aged 55–75 years at the time of baseline LDCT who currently or formerly smoked.	Total (*N* = 6036)
					Native Hawaiian/Pacific Islander and American Indian (*n* = 134)
Kee, 2020 [37]	2018	USA (National)	The 2018 BRFSS data and measured self‐reported annual LDCT imaging for lung cancer	Asymptomatic individuals aged 55–80 with 30 pack‐years smoking history who currently smoke or quit within the last 15 years	224 679 of 1 273 013 USPSTF criteria—eligible smokers reported annual LDCT screening
Rustagi, 2023 [38]	2017–2020	USA (sub‐national)	Quantitative	The USA military veterans aged 50–79 years and have a history of 30 pack‐years tobacco smoking, current smokers or quit smoking within the past 15 years and have not been diagnosed with lung cancer	3376 (71 AI/AN, 2 NH/PI, 2987 Non‐Hispanic White, 112 Non‐Hispanic Black, 3 Asian, 32 Other, 73 Multiracial, 35 Hispanic, 61 Missing)
			Population‐based cross‐sectional study		
Walker, 2021 [28]	2020	Canada (sub‐provincial)	Quantitative	Persons aged ≥ 55 years who visited six hospitals having LCS programmes across Ontario and have ≥ 2% risk of developing lung cancer in the coming 2 years.	3178 based on the 2020 LCS participation (90 First Nations and Inuit, 84 Métis and other Indigenous peoples, 2719 non‐Indigenous, 257 missing)
			Hospital‐based administrative database		
Gould, 2017 [27]	2013–2016	USA (sub‐national)	Quantitative	Four healthcare systems having LCS programmes	3822 participants screened in four healthcare systems (48 Native American/Alaskan/Pacific Islander, 2640 Non‐Hispanic white, 152 Asian, 332 Black, 314 Hispanic, 50 Other, 286 Unknown)
			Population‐based electronic medical records review		
Oshiro, 2022 [30]	2015–2019	USA (state)	Quantitative	Persons aged 55–79 years, cancer‐free or past at least 5 years of any lung cancer diagnosis and treatment and have a history of 30 pack‐years tobacco smoking, current smoker or quit smoking within the past 5 years.	1030 (186 Native Hawaiian, 63 Pacific Islander, 381 Non‐Hispanic White, 34 Chinese, 99 Filipino, 146 Japanese, 33 Korean, 88 Other)
			Population‐based data derived from electronic medical record		
Aredo, 2022 [31]	1993 to 2017	USA (sub‐national)	Quantitative	Persons with a smoking history who were diagnosed with incidental lung cancer from 1993 to 2017, identified by cancer registry linkage.	5900 (533 Native Hawaiian, 1579 White, 1660 African American, 1328 Japanese American, 800 Latino)
			Population‐based prospective cohort study		
Liu, 2022 [26]	2021–2022	USA (sub‐national)	Quantitative	Participants responded to questions related to the 2013 USPSTF LCS eligibility criteria and recommendations.	96 078 (2028 AI/AN, 288 NH/PI, 75 628 White, 2105 Asian, 10 782 Black or African American, 2825 Other Race, 2422 unknown/missing)
			Population‐based cross‐sectional study		
Narayan, 2021 [29]	2019	USA (sub‐national)	Quantitative	Participants aged 50–79 years and have not been diagnosed with lung cancer and self‐reported current or history of smoking.	77 689 based on the revised USPSTF criteria (1338 AI/AN, 402 Asian or Pacific Islander, 67 454 White, 1968 Hispanic, 3854 African American, 1425 Other)
			Population‐based cross‐sectional study		
Perez, 2022 [19]	2022	USA (national)	Quantitative	The evidence base for 10 various guidelines (e.g., American Society of Clinical Oncology, National Comprehensive Cancer Network, American College of Chest Physicians) to provide statements about LCS using the USPSTF guidelines.	Nine randomised controlled trial evidence
			Review of randomised controlled trial studies that were used to develop the LCS guideline (USPSTF).		
McLeod, 2020 [7]	2011	New Zealand (national)	Quantitative	Participants aged 55–74 years and have a history of smoking 30 pack‐years or quit smoking within the past 15 years.	Not reported, but the study participants were sourced from previous intervention studies.
			Intervention		

Abbreviations: AI/AN = American Indian/Alaska natives; aOR = adjusted odds ratio; LDCT = low‐dose computed tomography; LCS = lung cancer screening; NH/PI = native Hawaiian or other Pacific Islander; USA = the United States of America; USPSTF = the United States Preventive Services Task Force.

^a^ The geographical coverage of the studies within the country was grouped as the national level, sub‐national (covering more than one administrative unit, i.e., states, regional administrative units, province), state (conducted in a single administrative unit, i.e., state, regional administrative unit, province) and sub‐state (conducted in part of administrative units, i.e., states, regional administrative units, province).

^b^ Included the list of reported study designs and data sources.

^c^ The population included age group and sex of participants if reported by the article.

The quality of the included studies was assessed (Table S2), and most presented well‐defined research questions and possessed data suitable for addressing these inquiries and utilised appropriate sampling strategies [7, 26, 27, 28, 29, 30, 31, 32, 33, 34, 35, 36, 37, 38, 39, 40, 41, 42]. Most had a representative sample from the target population (*n* = 15) [26, 27, 28, 29, 30, 31, 32, 34, 35, 36, 37, 38, 39, 40, 42].

The key findings are structured to firstly present the cost‐effectiveness of LCS, establishing its importance, followed by an examination of participation rates, as determining the extent of screening participation is crucial to evaluating how widely and inclusively these benefits are being realised. Finally, an exploration of the barriers and facilitators of LCS is provided to identify the factors that either encourage or hinder participation, an insight into ensuring that the potential benefits of LCS are fully realised across various groups of people.

### 3.2 Cost‐Effectiveness of LCS: Māori (New Zealand) Experience

Two studies assessed the cost‐effectiveness of LCS among Māori and non‐Māori in New Zealand [7, 34]. The first study found LCS to be cost‐effective for both groups aged 55–74 years with a smoking history. Specifically, the incremental cost‐effectiveness ratios (ICERs) ranged from NZ$24 700 per Health Adjusted Life Year (HALY) (95% UI NZ$19 900 to NZ$30 400) for Māori females up to NZ$39 100 per HALY for non‐Māori males (95% UI NZ$31 400 to NZ$48 900), with health gains being twice as high for Māori females and 25% higher for Māori males compared to their non‐Māori counterparts [7]. The second study also reported lower screening costs for Māori ($570) compared to those for non‐Māori ($3915), with an ICER of $NZ101 000 for Māori and $NZ171 000 for non‐Māori, indicating greater cost‐effectiveness for Māori, particularly for Māori females aged 70–74 [34] (Table 2).

**TABLE 2:** Key findings on cost‐effectiveness, participation rates, facilitators and barriers of lung cancer screening.

ID	Key findings
Anderson, 2023 [39]	Barriers and facilitators
	Related to the barriers and facilitators of LCS, five themes were identified: fear, trust, approach to healthcare (reactive, proactive and traditional knowledge), optimism/openness to screening and determinants of LCS (insufficient knowledge about and inadequate resources/capacity to screening).
	The facilitators for LCS include patients and HCPs recognising that traditional Indigenous practices may complement Western medicine. Some participants connect well‐being to future generations, enhancing their health practices, including screening. Many AI/AN focus group participants were receptive to LCS, and some of the patients' interest grew after receiving health education on the screening. Furthermore, trust in their local clinic or provider appeared to be a significant factor in encouraging adherence to health service recommendations, including Lung Cancer Screening (LCS). Furthermore, healthcare provider participants collectively agree that lung cancer is a significant issue for American Indian and Alaska Native peoples, which could facilitate participation in LCS.
	The barriers for LCS include fear of the screening process, worries of being diagnosed with cancer or unpreferred ways of communicating the screening results (most patients prefer written communication with postal or electronic mail), negative attitudes to screening, mistrust and/or negative previous experiences with the health system, and limited knowledge regarding the LCS programme or the screening access. In addition, patients reported limited resources and/or capacity to conduct the LCS, including limited time and transportation options, low insurance coverage and childcare facility, inadequate resources to support throughout the screening process. The healthcare providers also reported insufficient time for screening, limited health promotion and education materials, and limited resources to identify eligible participants, ordering or referring screening.
Colhoun, 2024 [40]	Facilitators for LCS
	Positive attitudes towards LCS (personal experience, being informed, Whānau–hapū–iwi‐focused, A new lease on life)
	○ Māori participants expressed enthusiasm for LCS and engaged actively with the Kaupapa framework.
	○ They were optimistic about the programme contributing to the well‐being of future generations and showed concern for the welfare of their whānau.
	○ Participants appreciated the chance to become better informed about lung cancer and its screening, particularly regarding the associated risks and benefits.
	○ They were hopeful that early detection could offer them a new opportunity at life by catching the disease in its earlier stages.
	2 Practical support
	○ Assistance with transport was seen as essential, whether through taxi vouchers, parking passes or transportation to screening or mobile units in rural areas.
	○ The idea of a ‘one‐stop shopping’ was popular, with participants advocating for time off work and the ability to address multiple health needs in one visit.
	3 Kaupapa Māori approaches
	○ Culturally safe practices with a focus on Kaupapa Māori were deemed critical.
	○ Participants valued family‐centred processes, such as the ability to bring whānau members along and allowing children at appointments.
	○ They also appreciated having a culturally sensitive and supportive navigator to guide them through the process.
	○ Communication rooted in Te reo Māori and tikanga was suggested, though participants acknowledged that not all eligible individuals may resonate with or feel confident in these due to the impacts of colonisation.

	○ Whakawhanaungatanga (relationship building) was a core component, with participants suggesting that facilitators take time to build meaningful connections both at the outset and throughout the screening process.
	○ Trust and the friendly nature of the healthcare providers were also emphasised as important factors.
	4 Clear and effective communication
	○ Culturally relevant, clear communication that avoided clinical jargon and offered understandable explanations of risk was viewed as enabling.
	○ Participants expressed a desire for reassurance that participating in screening did not equate to a death sentence and that early detection could lead to curative outcomes.
	5 Programme messaging, awareness and role models
	○ Messaging focused on whānau, such as a ‘by Māori, for Māori’ approach to LCS, was seen as supportive, with careful attention to word choice being important. Strong yet encouraging language was necessary to motivate people to take screening invitations seriously, without discouraging them.
	○ Raising awareness among Māori communities about the importance of screening was crucial.
	○ Participants suggested that the broader community could model screening behaviour, thereby encouraging participation in LCS.
	6 Influences on decision‐making (information, relationships with doctors, Whānau decision‐makers, autonomy, source of invitation)
	○ Quality information, existing relationships with general practitioners (GPs), input from key whānau decision‐makers, autonomy in decision‐making and the source of the invitation could all affect the decision to participate in LCS.
	○ Some participants preferred to receive an invitation to LCS from their GP, rather than through an impersonal letter or phone call.
	○ The involvement of whānau decision‐makers was essential, with participants suggesting that family units and key decision‐makers should be targeted in order to encourage broader participation in LCS.
	Barriers to LCS
	Fear of the disease and negative past experiences with the health system (fear of the disease, prior negative experiences, distrust in relationships, negative messages spreading through word of mouth)
	○ Some participants expressed reluctance towards LCS, primarily driven by fear of the disease and negative past experiences with the healthcare system and screening processes. For some, their whānau members had not survived lung cancer, adding to their fear.
	○ There was a general sense of distrust in the health system, even extending to relationships with their own doctors.
	○ Participants also pointed out the risk of negative experiences being spread through word of mouth, which could deter others from participating.
	2 Other barriers, including access issues (cost, transport, time constraints due to Whānau/Work commitments, stigma, inconvenience)
	○ Access challenges were a major barrier for Māori participants when considering LCS. These challenges included financial costs, the time required for appointments and difficulties with transportation.
	○ Some participants highlighted that getting time off work to attend the screening was particularly difficult, as was finding suitable childcare arrangements.
	○ The inconvenience of needing to attend multiple appointments and make repeated visits to the hospital also acted as a deterrent.
○ Additionally, the stigma associated with smoking and its link to lung cancer further contributed to participants' hesitation regarding screening.
Welch, 2024 [41]	Barriers to LCS
	Limited awareness of LCS
	○ 57% of participants (33 out of 58) were unaware of LCS, and 67% (37 out of 58) did not know the eligibility criteria.
	○ Participants with the highest awareness of lung cancer risk factors often had personal or familial experiences with lung disease or knew community members affected by it.
	○ Some shared that fatalistic attitudes towards lung disease affected their family members, but those with personal connections advocated for screening.
	○ Opinions varied on whether lung cancer was seen as a significant concern in their communities, often influenced by personal experiences.
	○ One participant highlighted concern due to uranium mine exposure, while another noted a lack of awareness of LCS until the death of a community elder from lung cancer.
	○ Despite limited knowledge and participation in LCS, direct experiences with the disease spurred efforts to address lung cancer risks.
	2 Limited patient–physician conversations
	○ Participants mentioned that healthcare providers often discussed nicotine cessation but did not extend these discussions to LCS.
	○ After learning about the importance of LCS, many felt their previous interactions with physicians were insufficient.
	3 Lack of educational, diagnostic and screening resources
	○ Participants noted the absence of diagnostic tools and LCS resources at local health facilities.
	4 Mistrust of medical institutions
	○ Widespread mistrust towards academic and medical institutions was identified, partly due to inadequate discussions with clinicians and the lack of educational resources.
	○ This mistrust was linked to historical trauma.
	Facilitators to LCS (related to American Indians/Alaska natives adult health‐seeking practices)
	○ Available Health Resources and Programmes: Participants highlighted various trusted sources of health information, including tribal clinics and health fairs.
	○ Many participants sought health advice through the Internet, physicians or tribal health workers.
	○ After learning about LCS benefits, many participants expressed willingness to travel for screening, despite potential challenges. One mentioned, ‘Our clinic has drivers and more vehicles for transporting community members to specialists, though travelling out of state would be more difficult’. Participants listed existing resources like medical transportation and elder programmes to assist with appointments.
	○ Willingness to promote LCS education: Participants emphasised the importance of educational initiatives to raise awareness about lung cancer, especially related to tobacco use. They advocated for bringing this knowledge back to their communities and promoting workshops to enhance the understanding of LCS.
Pena, 2023 [36]	Access to LCS centres
	The authors identified significant barriers to lung cancer screening (LCS) access for American Indian/Alaska Native communities. Among LCS centres within 200 miles, only 26.9% were ACR accredited, and within 50 miles, just 31.5% had accreditation. American Indian/Alaska Native tribes in counties with persistent adult poverty (PPC‐A) and persistent child poverty (PPC‐C) designations were less likely to have LCS centres nearby, and even fewer had ACR‐accredited centres. Distance was a major barrier, with American Indian/Alaska Native communities facing limited access to nearby, accredited LCS centres, particularly in areas with poorer health infrastructure
Tsosie, 2024 [42]	Barriers to LCS
	Negative interactions with the healthcare system, whether related to cancer or not, led many participants to feel that AI/AN individuals receive lower‐quality care, fostering hesitation towards seeking services like LCS.
	Key barriers cited were perceived or actual costs and transportation challenges. One participant shared, ‘I've never requested [LCS] because of the fear of the cost’.
	Complex relationships with tobacco use influenced both LCS participation and cessation behaviours.
	Most participants had limited knowledge or experience with LCS, contributing to low engagement.
	Fear of screening procedures or potential cancer diagnoses discouraged some from seeking LCS, with the fear of cancer and discomfort leading them to avoid screenings.
	Twenty‐eight percentage reported experiencing discrimination in healthcare settings.
	Facilitators to LCS
	Participants had positive experiences with Indigenous‐centred care in community settings, which stood in contrast to care at external facilities.
	Most participants viewed LCS positively, recognising its importance for maintaining their health and that of their families.
	Family and community support were key facilitators for getting screened.
	The majority (91%) preferred receiving healthcare information and services in environments designed by and for AI/AN individuals.
Dignan, 2024 [33]	The Barriers to LCS
	Participants provided different barriers to LCS among participants. Key reasons for not undergoing low‐dose CT (LDCT) screening included the absence of a provider recommendation, feeling they were not at risk for lung cancer, distance to screening facilities, costs and fear of potential results.
	LCS participation rates
	During community education sessions, 2.4% of participants reported ever having a low‐dose CT scan (LDCT), which increased to 9.6% at the 6‐month follow‐up (*Z* = −5.5, *p* < 0.0001).
	Between July 2018 and June 2022, 2829 patients underwent LDCT screening at participating centres. This represented a 90.9% increase in LDCTs in year 4 compared to the 2017 baseline (from 640 to 1066). The primary reason cited for obtaining an LDCT was provider recommendation (88%, *n* = 962), with other factors including personal recommendations, educational sessions and mass media exposure.
Rustagi, 2023 [38]	Participation focused:
	Compared to Non‐Hispanic White (aOR 95% CI), AI/AN were 88% less likely to participate in LCS participation (aOR: 0.12 (0.03–0.44)).^a^
Walker, 2021 [28]	Participation focused:
	In 2020, 5.5% of the Canadian Indigenous peoples had LCS in the Ontario LCS centre, and this figure is comparable to Indigenous peoples' proportion (above 5%) on the national census estimate for 2021 [43]. However, the estimate was based on one LCS programme site and could not represent the general population, specifically Indigenous peoples, who are more likely to live far from the LCS programmes.
Gould, 2017 [27]	Participation focused:
	Compared to the general population, 1.3% of Native American/Alaskan/Pacific Islanders were screened, yet this figure is lower than these populations' proportion (above 2.4%) based on the national census estimate in 2017 [45].
Oshiro, 2022 [30]	Participation focused:
	There was a significant difference regarding LDCT order across sex within race/ethnic groups (*p* < 0.001). A higher proportion of Native Hawaiian women have LDCT orders than their Native Hawaiian men counterparts, while in other races/ethnicities, the proportion of LDCT orders was higher among men than women.
	Of those with LDCT screening orders, a 14%–15% screening completion rate gap between race/ethnic groups was reported. However, the completion rate of Native Hawaiian (80%) or Pacific Islanders (79%) was similar to that of Non‐Hispanic White (80%).
Aredo, 2022 [31]	Guideline focused:
	There was a significant difference across race/ethnic groups according to the 2021 USPSTF LCS criteria (*p* < 0.001). The percentage of Native Hawaiian (56.7%) eligible for LCS according to the 2021 USPSTF criteria was slightly higher than Whites (49.6%).
Liu, 2022 [26]	Guideline focused:
	Not fulfilling any of the 2013 USPSTF LCS eligibility criteria recommendations was higher among AI/AN (OR 95% CI, 0.95%: 1.43 (0.61–3.33)) or NH/PI (2.79 (0.55–14.09)) compared to Whites, yet this difference was not significant.
	LCS rate difference between eligible screened and eligible unscreened was higher among AI/AN (0.95%) or NH/PI (0.06%) than Whites (−2.67%). However, the LCS rate difference between ineligible screened and ineligible unscreened was lower among AI/AN (−0.84%) or NH/PI (−0.18%) than Whites 1.54%.
Narayan, 2021 [29]	Guideline focused:
	Compared to Whites, AI/AN was less likely to be eligible for LCS, yet the difference was not statistically significant (OR 95% CI: 0.84 (0.62–1.15)).^b^
Perez, 2022 [19]	Guideline focused:
	The guidelines related to LCS were developed based on nine randomised controlled trial evidence, yet race/ethnicity‐related evidence was reported by only two randomised controlled trials, and none of them has evidence regarding Indigenous peoples.
McLeod, 2020 [7]	Cost‐effectiveness focused:
	LCS was cost‐effective for Māori and non‐Māori participants aged 55–74 years with a smoking history (NZ$45 000 per Healthy Adjusted Life Years (HALY)). Specifically, the incremental cost‐effectiveness ratios (ICERs) ranged from NZ$24700 per HALY (95% UI NZ$19 900 to NZ$30 400) for Māori females up to NZ$39 100 per HALY for non‐Māori males (95% UI NZ$31 400 to NZ$48 900).
	In addition, the size of health gains as a result of LCS was higher among Māori and non‐Māori participants, with two times higher among Māori females and 25% times higher among Māori males compared to non‐Māori females and males, respectively.
Jaine, 2018 [34]	Cost‐effectiveness focused:
	The total cost of screening was $570 (462–681) for Māori and $3915 (3189–4670) for non‐Māori.
	The incremental cost‐effectiveness ratio was $NZ101 000 (62 000–162 000) for Māori and $NZ171 000 (94 000–296 000) for non‐Māori, indicating that low‐dose computed tomography (LDCT) is more cost‐effective for Māori compared to that for non‐Māori.
	The most favourable incremental cost‐effectiveness ratio was $NZ77 000 (US$52 000) for Māori females aged 70–74, while the least favourable was $NZ211 000 (US$142 000) for non‐Māori females aged 55–59.
Choi, 2023 [32]	Guideline focused
	The 2021 USPSTF criteria revealed a significant disparity, with Native Hawaiians having a 17% lower E‐I ratio compared to White individuals (16.8 vs. 20.3; *p* < 0.001). Using risk‐based screening (PLCOm2012‐Update with 6‐year risk ≥ 1.3%), this gap decreased to 10% (16.6 vs. 18.4; *p* < 0.001).
	Regarding eligibility, the USPSTF 2021 criteria showed a 5% disparity between Native Hawaiians and Whites (25.1% vs. 30.2%). Under risk‐based screening, this difference was reduced to 3% (24.7% vs. 27.4%).
Kim, 2022 [35]	Participation focused
	Among those with a negative baseline screen, adherence to annual lung cancer screening was 76% for American Indian/Alaska Native peoples, 79.3% for Native Hawaiians/Pacific Islanders and 56.3% for Whites. For those with a positive baseline screen, adherence was 75% for American Indian/Alaska Native peoples, 100% for Native Hawaiians/Pacific Islanders and 70.3% for Whites.
Kee, 2020 [37]	Participation focused
	The authors reported that 1.83% (0.82%–2.83%) of American Indians/Alaska Natives were eligible for screening among all LDCT‐eligible individuals, with only 1.09% (0.00%–2.18%) actually receiving it. In contrast, 85.96% (81.45%–90.46%) of Whites were eligible for screening, with 84.12% (71.09%–97.14%) receiving it. The proportion of American Indian/Alaska Native people eligible for LCS and receiving the screening was relatively small compared to their 2.2% representation in the total population in 2022 [44].

Abbreviations: AI/AN = American Indian/Alaska Natives; aOR = adjusted odds ratio; LDCT = low‐dose computed tomography; LCS = lung cancer screening; NH/PI = Native Hawaiian or other Pacific Islander; USA = the United States of America; USPSTF = the United States Preventive Services Task Force.

^a^ Adjusted for sex, marital status, BMI, education, smoking history in pack‐years, health insurance status, receipt of influenza vaccine in prior 12 months, difficulty paying for medical care, diagnosis of chronic obstructive pulmonary disease, personal history of non‐lung cancer and survey year.

^b^ Model adjusted for age, education level, income category, married or domestic partnership status, sex, having at least one personal doctor, insurance status and employment status.

### 3.3 Participation in LCS Service

Limited evidence exists regarding Indigenous Peoples' participation in LCS. In the USA, among 3376 US military veterans, American Indians and Alaska Natives had an 88% lower LCS participation rate (4.7%) compared to Non‐Hispanic Whites (21.7%) (aOR: 0.12, 95% CI: 0.03–0.44) [38]. In 2020, 5.5% of the total population screened in Ontario LCS centres in Canada were Indigenous [28], exceeding their proportion in the 2021 census estimate (2.9%) [43]. This screening rate should be interpreted cautiously due to unknown specific LCS site locations and varying Indigenous population distributions, such as 17% in northern Ontario [43]. Furthermore, in a US study, participants with LDCT prescriptions reported comparable completion rates between Native Hawaiians and non‐Hispanic White peoples, with both groups achieving an 80% completion rate [30] (Table 2). Additionally, one study found that 76% of American Indians/Alaska Natives individuals with a negative baseline adhered to annual LCS, compared to 79.3% of Native Hawaiians/Pacific Islanders and 56.3% of Whites, while for positive baseline screens, adherence was 75% for American Indian/Alaska Native, 100% for Native Hawaiians/Pacific Islanders and 70.3% for Whites [35]. Another study reported that 1.83% of American Indians/Alaska Natives were eligible for screening among all LDCT‐eligible individuals, with only 1.09% receiving it [37]. The proportion of American Indians/Alaska Natives eligible for and receiving LCS was relatively small compared to their 2.2% share of the total population in 2022 [44]. Furthermore, another study revealed that LCS participation among American Indians increased from 2.4% at community education sessions to 9.6% after 6 months [33] (Table 2).

### 3.4 Facilitators and Barriers to LCS

Studies comprehensively explored possible barriers and facilitators to LCS participation experienced by Indigenous peoples (such as American Indians/Alaska Natives and Māori peoples) (Table 2).

**FIGURE 2:**
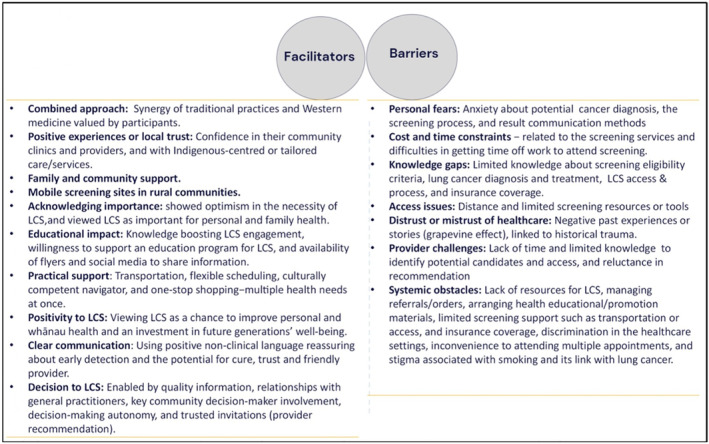
Barriers and facilitators of lung cancer screening participation [33, 39, 40, 41, 42].

Multiple facilitators of LCS participation among Indigenous peoples were consistently identified across studies [39, 40, 41, 42]. For American Indians and Alaska natives, facilitators included belief in the synergy between traditional and Western medicine, trust in their local clinics and healthcare providers, LCS acceptance, health education strategy implementation, and personal health and health gains being viewed as a commitment to future generations—driving behaviours like cancer screening [39] (Figure 2). Tsosie et al. also found positive experiences with Indigenous‐centred or tailored care, family and community support, LCS's perceived importance for personal and family health, and healthcare information and services designed specifically by and for American Indians and Alaska Native peoples [42]. Welch et al. [41] highlighted additional facilitators for LCS among American Indians and Alaska Natives communities such as trusted health information through tribal clinics, health fairs, the Internet and tribal health workers. Social media, flyers and elder programmes support in sharing information or health education. Many were willing to travel for screening after learning about LCS benefits, with tribal resources like medical transportation and elder programmes aiding access, and support for more educational efforts and programming for LCS. Among Māori peoples, facilitators included positive views of LCS as a way to improve personal and whānau health, with hopes for healthier future generations. Practical support, such as transport and flexible scheduling, mobile sites in rural communities, one‐stop shopping—multiple health needs at once, programme facilitators' strong relationship with the screening candidates, trust and friendliness of providers, and a Kaupapa Māori approach ensuring cultural safety, whānau involvement and culturally competent navigators, culturally responsive clear non‐clinical communication about early detection and cure potential, reassured participants. Messaging ‘by Māori, for Māori’, raised awareness using positive language [40]. The decision to participate in LCS was facilitated by the quality of information (detailed and factual), relationships with general practitioners, involvement of key whānau decision‐makers, autonomy over the decision‐making process and invitations from trusted sources like their general practitioners [40]. Furthermore, provider recommendations, educational sessions and mass media exposure were noted as reasons for obtaining LCS [33] (Figure 2).

The barriers to LCS participation for Indigenous peoples (American Indians/Alaska Natives, Māori) were discussed across studies and included factors at the individual, health professional and system levels (Table 2). Individual‐level factors included fears of receiving a cancer diagnosis or outcome [33, 39, 40, 41, 42], time constraints, limited knowledge about lung cancer and screening processes [39, 42], low awareness (with 57% unaware of LCS and 67% unfamiliar with its criteria) [41], feelings of not being at risk for lung cancer [33], financial costs including insurance coverage, general distrust or mistrust of healthcare [39, 40] and negative past experiences or interactions with the healthcare system [39, 40], Additionally, the ‘grapevine’ effect, where negative stories about healthcare spread within the community, was a concern [39, 40]. Healthcare professionals faced challenges such as insufficient time, limited knowledge for identifying eligible individuals and access issues to screening programmes [39]. Patient–physician conversations often focused mainly on smoking cessation without addressing LCS, which participants found insufficient [41], compounded by the absence of a provider recommendation [33] (Figure 2). Systemic barriers involved limited resources for identifying eligible candidates, implementation issues, managing orders or referrals, health promotion/education [39], costs or time to attend appointments and transportation difficulties [33, 39, 40, 41, 42]. Distance was a major barrier, especially for American Indians and Alaska Native communities, with limited access to nearby, accredited LCS centres, particularly in areas with poorer health infrastructure [36]. Clinics also lacked educational materials and screening tools, limiting access to LCS resources [41]. The inconvenience of multiple hospital visits [39, 40] and the stigma associated with smoking and its link to lung cancer further discouraged participation [40], experiencing discrimination in healthcare settings [42], and there was a need for support throughout the screening process, such as addressing transportation challenges, health insurance coverage and childcare facilities [39, 40] (Figure 2).

Additionally, LCS eligibility guidelines or criteria are considered major system‐level barriers, as Indigenous peoples may have been excluded due to criteria that are not tailored to the specific population likely as a result of an uninclusive guidelines development process. Several studies reported on LCS guideline eligibility for Indigenous peoples. Five studies discussed LCS eligibility guidelines or criteria for Indigenous peoples [19, 26, 29, 31, 32]. Choi et al. found that both the 2021 United States Preventive Services Task Force (USPSTF) criteria and the Prostate, Lung and Ovarian Cancer Screening Trial 2012 (PLCOm2012) Update screening method revealed a significant disparity in eligibility‐incidence ratios, with Native Hawaiians consistently having lower ratios than White individuals (16.8 vs. 20.3 and 16.6 vs. 18.4, respectively; both *p* < 0.001), and a 5% eligibility gap between the groups under the USPSTF criteria (25.1% vs. 30.2%) [32]. Another study exploring the evidence foundation for LCS guidelines examined numerous trials as a source of information for developing LCS guidelines. Yet, none of the trials reported the involvement of Indigenous peoples in authoring the guidelines [19] (Table 2). Inconsistent evidence exists regarding the screening eligibility of Indigenous peoples in LCS guidelines. According to the 2021 USPSTF criteria, LCS eligibility significantly differed across populations, with a slightly higher proportion of Native Hawaiians (56.7%) eligible than non‐Indigenous White Americans (49.6%) [31]. However, two other studies did not show variations in screening eligibility by race/ethnicity [26, 29]. Although not statistically significant, a lower proportion of American Indians/Alaskan Natives were eligible for LCS according to the USPSTF guideline compared to White populations (odds ratio: 95% CI: 0.84 [0.62–1.15]) [29].

Two studies [39, 42] suggested a suite of strategies to overcome the identified barriers. These strategies included the development of culturally relevant multimedia education materials, organising community screening events and offering referral site options. Additionally, patient navigation was preferred for addressing individualised barriers, helping with paperwork, scheduling, reminders and facility access (Table S3).

## 4 Discussion

LCS screening is an evolving initiative, with only 10 countries worldwide currently implementing it at the national and/or regional level [5]. To our knowledge, this is the first systematic review to explore the LCS among Indigenous peoples globally, specifically focusing on its cost‐effectiveness, participation, and associated barriers and facilitators. While LCS was reported as a cost‐effective approach for Māori people [7, 34], its effectiveness remains under ongoing evaluation. In relation to LCS participation, for instance, American Indians/Alaska Natives had an 88% lower LCS participation rate (4.7%) compared to non‐Hispanic Whites (21.7%) [38]. The lower participation rate among Indigenous peoples, such as American Indians/Alaska Natives and Māori, occurs due to multiple factors at the individual, healthcare professional and system levels. Barriers include the low awareness of LCS or the eligibility criteria, fear of the screening process or cancer diagnosis, mistrust of the healthcare system, negative experience or discrimination in the healthcare settings, cost and time constraints, limited transportation options or access, and insufficient resources such as screening tools, and eligibility criteria that are not inclusive or tailored to Indigenous populations [33, 39, 40, 41, 42]. Notably, these barriers to LCS have been documented in studies examining other cancer screening programs or services [12, 13, 46, 47, 48]. However, studies also identified factors that could facilitate LCS, such as positive views of LCS, trust in local clinics (Indigenous‐centred care) and providers, trusted invitations such as from their general practitioners, clear communication, family and community support, practical support such as transportation or flexible scheduling, availability of culturally competent navigators, detailed and factual information and willingness to be screened after health education. Overall, caution is needed when translating the findings of this review, as the evidence was drawn from a limited number of countries, and the studies from these countries were not conducted at a nationwide level.

Eligibility for LCS is determined by guidelines developed through inclusive and collaborative research involving various stakeholders. However, the inclusion of Indigenous peoples in the trials that informed the development of these guidelines was minimal or non‐existent [19]. Furthermore, the eligibility‐to‐incidence ratio for Indigenous peoples, such as Native Hawaiians, is reported to be lower than that for White individuals, suggesting that Native Hawaiians are less likely to meet the eligibility criteria despite experiencing higher rates of cancer [32]. To address these, the development of LCS eligibility guidelines and LCSPs should adopt an evidence‐based approach that considers the needs of diverse populations, including Indigenous peoples [49]. In this context, co‐design principles are essential; they go beyond best practices by emphasising the active involvement of stakeholders, Indigenous community members and individuals with lived experience of lung cancer from the outset [50]. Culturally appropriate co‐design processes are necessary to build trust and create an equitable LCS pathway for Indigenous populations. Efforts are in progress to co‐design LCS for Indigenous peoples. In Aotearoa New Zealand, a randomised trial protocol is set to assess optimisation options for LCS among Indigenous populations in 2024. This includes evaluating the relative importance of LCS invitations from primary care physicians compared to centralised screening services while ensuring the evidence‐based, safe implementation and maintenance of the LCSP [14]. In Australia, a trial is underway, assessing the feasibility of culturally tailored approaches for Indigenous Australians, drawing on local resources from the Queensland Lung Cancer Screening Study (QLCSS) and international resources from the International Lung Screening Trials (ILST). The aim is to establish a culturally safe LCSP that actively and respectfully recognises, values and incorporates the cultural beliefs, practices and perspectives of individuals and communities by 2025 [51, ^52^].

Evidence regarding barriers and facilitators of LCS among Indigenous peoples is limited, necessitating further research to gain a comprehensive understanding of these factors. Some barriers and facilitators identified in this review align with those previously reported for Indigenous Australians in other cancer screening programmes, such as for bowel, cervical and breast cancers [46, 47, 48]. This underscores the importance of leveraging knowledge from existing cancer screening programmes. Barriers reported include the low awareness of LCS, mistrust of the health system, discrimination in the healthcare settings and unmet needs related to the capacity of the LCSP. These issues are consistent with the findings from previous studies focused on other minority racial or ethnicity groups, such as Non‐Hispanic Black Americans [12, 13]. The barriers to LCS among Indigenous peoples may be linked with various critical and overlapping factors, including racism, colonisation, a lack of cultural competence in healthcare services, language barriers, ineffective communications, inadequate access to health services and limited health education [53, 54]. For example, LCSPs are more likely to be located in high‐ranking urban health facilities, which generally have more workforce and material resources [55]. This creates a significant discrepancy in access to LCS service access. Understanding the complexities of these higher‐level health facilities presents a challenge for Indigenous peoples, who often reside in rural and remote areas [53, 54].

Strengthening efforts to reduce LCS‐related barriers for Indigenous peoples is essential for improving participation. Previous evidence has identified successful strategies to improve LCS participation among Indigenous peoples. These strategies include providing personalised health education and information resources for both LCS service users and providers, expanding mobile LCSPs and transportation options, fostering trust in LCSPs through collaboration with Indigenous communities, training and providing culturally competent screening services, enhancing the capacity of LCS facilities with workforce development, clinical and educational tools, and effective referral and follow‐up systems [54, 56]. The qualitative studies [39, 42] included in this review suggested multifaceted strategies to enhance LCS participation among Indigenous peoples. These approaches include the routine inclusion of LCS in healthcare, employing patient advocates, encouraging family and community support, leveraging technology and material to streamline the screening process, expanding mobile screening sites, establishing one‐stop shopping—multiple health needs at once—providing user‐friendly eligibility assessment tools for healthcare providers, ensuring availability of culturally competent navigators, flexible screening scheduling, recognising community‐specific barriers, scheduling referrals during clinic visits or ensuring flexible scheduling, implementing efficient screening methods with respect to patients' time and developing culturally relevant multimedia educational materials. Furthermore, expanding Indigenous‐controlled health facilities, such as Aboriginal Community‐Controlled Health Organisations [ACCHOs] in Australia, along with LCS programmes, should be prioritised for sustainable improvements in LCS participation, access and trust. These facilities offer culturally competent services, workforce development, ownership and self‐governance, and Indigenous solutions to challenges faced by these communities [56, 57].

### 4.1 Strengths and Limitations

This systematic review represents the first effort to synthesise evidence regarding Indigenous peoples' participation in LCSPs globally. The search terms were thoroughly developed based on published research and consultations with experts in Indigenous health. The search process was rigorous, encompassing various databases and extensive supplementary searches on Google Scholar, as well as forward and backward citation searches of included articles using the Citationchaser tool. However, this review faced some challenges. The review is limited to studies involving Indigenous peoples, primarily American Indians/Alaska Natives or Māori. As Indigenous peoples are not a homogeneous group, the findings may not be generalisable to other Indigenous populations. Additionally, inconsistencies in reporting Indigenous status presented a significant limitation; for example, three studies were excluded from this review because they did not differentiate between Indigenous peoples and other racial or ethnic groups [58, 59, 60], which created ambiguity in interpreting figures and led to their exclusion. This issue highlights the ongoing challenges in research reporting practices, despite longstanding recommendations for the separate reporting of Indigenous data. Effective implementation of this practice is evident in census data collection, as seen in the US census, which has consistently reported data for Native Hawaiians as a distinct group for over 20 years [61]. Lastly, some peer‐reviewed articles may have been overlooked due to publication in languages other than English.

## 5 Conclusions

This review highlights the need for further research to fully understand the landscape of LCS among Indigenous peoples. To enhance Indigenous peoples' participation in LCS, it is essential to strengthen initiatives for culturally appropriate and acceptable LCS programmes, improve health education, enhance facility capacities with adequate instruments and workforce, and expand access through funding while extending LCSPs to rural and remote areas. These strategies are fundamental to achieving equity in LCS services. For Australia and similar countries preparing for LCSPs, global evidence underscores the importance of identifying facilitators of LCS for Indigenous peoples and co‐designing LCS programmes with Indigenous communities. Furthermore, addressing multi‐level barriers by ensuring adequate resources and providing culturally tailored health education for both community members and health professionals is vital for fostering effective and inclusive LCSP for Indigenous peoples worldwide.

## Ethics Statement

Our study, involving a secondary analysis of prior works, was exempt from ethics review. The project team includes senior Indigenous researchers from Australia and Aotearoa New Zealand, and the research was conducted under their supervision and guidance.

## Conflicts of Interest

The authors declare no conflicts of interest.

## Supporting information


**Data S1.** Supporting Information.

## Data Availability

Data sharing is not applicable to this article as no new data were created or analyzed in this study.
