# Supplementary material for: Lung Cancer Screening Participation Among Indigenous Peoples Worldwide: A Systematic Review of Challenges and Opportunities
Source: Health Promot J Austr. 2025 Feb 24;36(2):e70001. doi: 10.1002/hpja.70001 (PMC11850951; doi:10.1002/hpja.70001)
Supplement: Supplementary file 1 — Data S1. Supporting Information. [file HPJA-36-0-s001.docx]

**Supplementary information**

**TABLE S1: Article search strategy using PubMed database.**

| ((("lung cancer screen*"[tiab] OR "lung cancer screening*"[tiab] OR "screening* lung cancer"[tiab] OR "pulmonary screening" [tiab])) OR ((("lung neoplasms"[Mesh Terms ] OR "lung cancer"[tiab] OR "Pleural Neoplasms"[Mesh Terms] OR "non-small cell lung cancer"[tiab] OR "small cell lung cancer"[tiab] OR "lung carcinoma*"[tiab] OR "lung blastoma"[tiab] OR "lung tumour"[tiab] OR "pulmonary neoplasm"[tiab] OR "bronchopulmonary cancer"[tiab] OR "lung malignan*"[tiab] OR "lung blastoma*"[tiab] OR "pneumoblastoma*"[tiab] OR "pleuropulmonary blastoma"[tiab] OR "pleural mesothelioma"[tiab] OR "Malignant Pleural Mesothelioma"[tiab] OR "bronchoalveolar cancer"[tiab] OR "lung nodule*" [tiab])) AND ((screen*[tiab] OR "mass screening"[MeSH Terms] OR "early detection of cancer"[MeSH Terms] OR "early diagnosis"[Tiab] OR "early discovery"[tiab])))) AND ("first nations"[All Fields] OR "american indian or alaska native"[MeSH Terms] OR "american indian or alaska native"[All Fields] OR "american native continental ancestry group"[All Fields] OR "oceanic ancestry group"[All Fields] OR "asian continental ancestry group" [All Fields] OR "indigenous"[All Fields] OR "health services, indigenous"[MeSH Terms] OR "indians, north american"[MeSH Terms] OR "north american indians"[All Fields] OR "aboriginal*"[All Fields] OR "inuit"[MeSH Terms] OR "inuit*"[All Fields] OR "metis"[All Fields] OR "samoa*"[All Fields] OR "american samoa"[MeSH Terms] OR "american samoa"[All Fields] OR "polynesia"[MeSH Terms] OR "polynesia"[All Fields] OR "maori*"[All Fields] OR "Native Hawaiian*"[All Fields] OR "Kanaka Maoli*"[All Fields] OR "aborigin*"[All Fields] OR "Torres Strait Islander*"[All Fields] OR "haida"[All Fields] OR "cree"[All Fields] OR "ojibwe"[All Fields] OR "anishnawbe"[All Fields] OR "anishinaabe*"[All Fields] OR "mohawk*"[All Fields] OR "dene"[All Fields] OR "algonquin*"[All Fields] OR "mississauga*"[All Fields] OR "seneca"[All Fields] OR "indigenous Canadians"[MeSH Terms] OR "indigenous Canadians" [All Fields] OR "Alaska Natives"[MeSH Terms] OR "Alaska Natives"[All Fields] OR "Indigenous Peoples"[MeSH Terms] OR "Indigenous Peoples"[All Fields] OR "Indians, South American"[MeSH Terms] OR "Indians, South American"[All Fields] OR "Australian Aboriginal and Torres Strait Islander Peoples"[MeSH Terms] OR "Australian Aboriginal and Torres Strait Islander Peoples"[ All Fields] OR "Indians, Central American"[MeSH Terms] OR "Indians, Central American"[All Fields] OR "Maori People"[MeSH Terms] OR "Asian American Native Hawaiian and Pacific Islander"[MeSH Terms] OR "Asian American Native Hawaiian and Pacific Islander"[All Fields] OR "Native Hawaiian or Other Pacific Islander"[MeSH Terms] OR "Native Hawaiian or Other Pacific Islander"[All Fields] OR "nunavut*"[tiab] OR "nunavik"[tiab] OR "torres strait*"[tiab] OR "eskimo*"[tiab] OR "native*"[tiab]) |
| --- |

**TABLE S2: Quality appraisal of the included studies using Mixed Methods Appraisal Tool (MMAT) (n=19)**

| **Article** | **Study designs** | **Screening questions**  **(for all types)^1^** | | **Qualitative** | | | | | **Quantitative** | | | | |
| --- | --- | --- | --- | --- | --- | --- | --- | --- | --- | --- | --- | --- | --- |
|  |  | **S1** | **S2** | **1.1** | **1.2** | **1.3** | **1.4** | **1.5** | **4.1** | **4.2** | **4.3** | **4.4** | **4.5** |
| **Gould, 2017 (1)** | Quantitative | Yes | Yes |  |  |  |  |  | Yes | Yes | Yes | Yes | Can’t tell |
| **Rustagi, 2022(2)** | Quantitative | Yes | Yes |  |  |  |  |  | Yes | Yes | Yes | Yes | Yes |
| **Walker, 2021 (3)** | Quantitative | Yes | Yes |  |  |  |  |  | Yes | Yes | Yes | Yes | Can’t tell |
| **Liu, 2022 (4)** | Quantitative | Yes | Yes |  |  |  |  |  | Yes | Yes | Yes | Yes | Yes |
| **Anderson, 2023 (5)** | Qualitative | Yes | Yes | Yes | Yes | Yes | Yes | Yes |  |  |  |  |  |
| **McLeod, 2020 (6)** | Quantitative | Yes | Yes |  |  |  |  |  | Yes | Yes | Yes | Yes | Yes |
| **Oshiro, 2022 (7)** | Quantitative | Yes | Yes |  |  |  |  |  | Yes | Can’t tell | Yes | Yes | Yes |
| **Aredo, 2021 (8)** | Quantitative | Yes | Yes |  |  |  |  |  | Yes | Yes | Yes | Yes | Yes |
| **Narayan, 2021 (9)** | Quantitative | Yes | Yes |  |  |  |  |  | Yes | Yes | Yes | Yes | Yes |
| **Perez, 2022 (10)** | Quantitative | Can’t tell | Can’t tell |  |  |  |  |  |  |  |  |  |  |
| **Colhoun, 2024 (11)** | Qualitative | Yes | Yes | Yes | Yes | Yes | Yes | Yes |  |  |  |  |  |
| **Welch, 2024 (12)** | Qualitative | Yes | Yes | Yes | Can’t tell | Yes | Yes | Yes |  |  |  |  |  |
| **Pena, 2023 (13)** | Quantitative | Yes | Yes |  |  |  |  |  | Yes | Yes | Yes | Yes | Yes |
| **Tsosie, 2024 (14)** | Qualitative | Yes | Yes | Yes | Yes | Yes | Yes | Yes |  |  |  |  |  |
| **Dignan, 2024 (15)** | Quantitative | Yes | Yes |  |  |  |  |  | Yes | Can’t tell | Yes | Can’t tell | Yes |
| **Jaine, 2018 (16)** | Quantitative | Yes | Yes |  |  |  |  |  | Yes | Yes | Yes | Yes | Yes |
| **Choi, 2023 (17)** | Quantitative | Yes | Yes |  |  |  |  |  | Yes | Yes | Yes | Yes | Yes |
| **Kim, 2022 (18)** | Quantitative | Yes | Yes |  |  |  |  |  | Yes | Yes | Yes | Yes | Yes |
| **Kee, 2020 (19)** | Quantitative | Yes | Yes |  |  |  |  |  | Yes | Yes | Yes | Yes | Yes |

Response: Yes, No, Can’t tell

^1^ Further appraisal may not be feasible or appropriate when the answer is ‘No’ or ‘Can’t tell’ to one or both screening questions.

S1. Are there clear research questions?

S2. Do the collected data allow to address the research questions?

1.1. Is the qualitative approach appropriate to answer the research question?

1.2. Are the qualitative data collection methods adequate to address the research question?

1.3. Are the findings adequately derived from the data?

1.4. Is the interpretation of results sufficiently substantiated by data?

1.5. Is there coherence between qualitative data sources, collection, analysis and interpretation?

4.1. Is the sampling strategy relevant to address the research question?

4.2. Is the sample representative of the target population?

4.3. Are the measurements appropriate?

4.4. Is the risk of nonresponse bias low?

4.5. Is the statistical analysis appropriate to answer the research question?

**Articles**

1. Gould MK, Sakoda LC, Ritzwoller DP, Simoff M, Neslund-Dudas C, Kushi LH, et al. Monitoring Lung Cancer Screening Use and Outcomes at Four Cancer Research Network Sites. Annals of the American Thoracic Society. 2017;14(12):1827-35
2. Rustagi AS, Byers AL, Brown JK, et al. Lung Cancer Screening Among U.S. Military Veterans by Health Status and Race and Ethnicity, 2017–2020: A Cross-Sectional Population-Based Study. AJPM Focus. 2023;2(2).
3. Walker MJ, Meggetto O, Gao J, Espino-Hernández G, Jembere N, Bravo CA, et al. Measuring the impact of the COVID-19 pandemic on organized cancer screening and diagnostic follow-up care in Ontario, Canada: A provincial, population-based study. Preventive medicine. 2021;151:106586.
4. Liu Y, Pan IWE, Tak HJ, Vlahos I, Volk R, Shih Y-CT. Assessment of Uptake Appropriateness of Computed Tomography for Lung Cancer Screening According to Patients Meeting Eligibility Criteria of the US Preventive Services Task Force. JAMA Network Open. 2022;5(11): e2243163-e.
5. Anderson MD, Pickner WJ, Begnaud A. Determinants of Lung Cancer Screening in a Minnesota Urban Indigenous Community: A Community-Based, Participatory, Action-Oriented Study. Cancer Prev Res (Phila). 2023;16(4):239-45.
6. McLeod M, Sandiford P, Kvizhinadze G, Bartholomew K, Crengle S. Impact of low-dose CT screening for lung cancer on ethnic health inequities in New Zealand: a cost-effectiveness analysis. BMJ Open. 2020;10(9): e037145.
7. Oshiro CES, Frankland TB, Mor J, Wong CP, Martinez YT, Aruga CKK, et al. Lung Cancer Screening by Race and Ethnicity in an Integrated Health System in Hawaii. JAMA Netw Open. 2022;5(1): e2144381.
8. Aredo JV, Choi E, Ding VY, Tammemägi MC, Ten Haaf K, Luo SJ, et al. Racial and Ethnic Disparities in Lung Cancer Screening by the 2021 USPSTF Guidelines Versus Risk-Based Criteria: The Multiethnic Cohort Study. JNCI Cancer Spectr. 2022;6(3).
9. Narayan AK, Chowdhry DN, Fintelmann FJ, Little BP, Shepard JO, Flores EJ. Racial and Ethnic Disparities in Lung Cancer Screening Eligibility. Radiology. 2021;301(3):712-20.
10. Perez NP, Baez YA, Stapleton SM, Muniappan A, Oseni TS, Goldstone RN, et al. Racially Conscious Cancer Screening Guidelines: A Path Towards Culturally Competent Science. Annals of Surgery. 2022;275(2):259-70.
11. Colhoun SR, Parker K, McCook S, et al. Perspectives of potentially eligible Indigenous Māori on a lung cancer screening programme: a qualitative study. *The New Zealand Medical Journal*. 2024;137(1593):45-55.
12. Welch AC, London SM, Wilshire CL, et al. Access to lung cancer screening among American Indian and Alaska Native Adults: a qualitative study. *Chest*. 2024;165(3):716-24.
13. Peña MA, Sudarshan A, Muns CM, et al. Analysis of geographic accessibility of breast, lung, and colorectal cancer screening centers among American Indian and Alaskan Native Tribes. *Journal of the American College of Radiology*. 2023;20(7):642-51.
14. Tsosie U, Anderson N, Woo N, et al. Understanding determinants of lung cancer preventive care in at-risk urban American Indians and Alaska Natives: A mixed-methods study. *Preventive Medicine Reports*. 2024;45:102822.
15. Dignan M, Cina K, Sargent M, et al. Increasing Lung Cancer Screening for High-Risk Smokers in a Frontier Population. *Journal of Cancer Education*. 2024;39(1):27-32.
16. Jaine R, Kvizhinadze G, Nair N, et al. Cost-effectiveness of a low-dose computed tomography screening programme for lung cancer in New Zealand. *Lung Cancer*. 2018;124:233-40.
17. Choi E, Ding VY, Luo SJ, et al. Risk model–based lung cancer screening and racial and ethnic disparities in the US. *JAMA oncology*. 2023;9(12):1640-8.
18. Kim RY, Rendle KA, Mitra N, et al. Racial disparities in adherence to annual lung cancer screening and recommended follow-up care: a multicenter cohort study. *Annals of the American Thoracic Society*. 2022;19(9):1561-9.
19. Kee D, Wisnivesky J, Kale MS. Lung cancer screening uptake: analysis of BRFSS 2018. *Journal of general internal medicine*. 2021;36:2897-9.

**TABLE S3: Strategies to overcome identified barriers, findings from the included qualitative studies.^1,2^**

| **Strategies proposed** | |
| --- | --- |
| **Healthcare professional participants' recommendations^1^** | Development of culturally relevant multimedia education materials |
|  | Community screening events |
|  | Provide comprehensive care incorporating spiritual and behavioural health elements to complement medical advice. |
|  | Casual interactions, such as booths |
|  | Ensuring inclusion of LCS in regular visits |
|  | Provide referral site options |
|  | User-friendly eligibility tools |
|  | Patient advocates |
|  | Rewards or support to encourage participation and improve access |
| **The study investigators’ recommendations^1^** | - Understand cultural values |
|  | - Promote health |
|  | - Schedule referrals during clinic visits |
|  | - Recognise community-specific barriers to LCS participation |
|  | - Implement efficient cancer screening processes that value patient time |
| **Interventions to Improve LCS^2^** | Patient navigation was favored to address individualized barriers, helping with paperwork, scheduling, reminders, and facility access. |
|  | Strong support for patient education and assistance throughout the care process. |
|  | Education on LCS that is tailored to the American Indian/Alaska Native peoples' experience, incorporating their culture, and expanding outreach beyond clinical settings. |

LCS: Lung Cancer Screening

1. Anderson MD, Pickner WJ, Begnaud A. Determinants of Lung Cancer Screening in a Minnesota Urban Indigenous Community: A Community-Based, Participatory, Action-Oriented Study. *Cancer Prev Res (Phila)*. 2023;16(4):239-45.
2. Tsosie U, Anderson N, Woo N, et al. Understanding determinants of lung cancer preventive care in at-risk urban American Indians and Alaska Natives: A mixed-methods study. Preventive Medicine Reports. 2024;45:102822.
